# A systematic review of how researchers characterize the school environment in determining its effect on student obesity

**DOI:** 10.1186/s40608-015-0045-5

**Published:** 2015-03-08

**Authors:** Kyle Turner, Charlie Foster, Steven Allender, Emma Plugge

**Affiliations:** Nuffield Department of Population Health, University of Oxford, Old Road Campus, Roosevelt Drive, Oxford, OX3 7LF United Kingdom; World Health Organization Collaborating Centre for Population Approaches to Non-Communicable Disease Prevention, University of Oxford, Oxford, UK; World Health Organization Collaborating Centre for Obesity Prevention, Deakin University, Burwood, 3125, Victoria Australia

**Keywords:** School, Childhood obesity, Environmental measurement

## Abstract

**Background:**

Obesity in early childhood is a robust predictor of obesity later in life. Schools provide unparalleled access to children and have subsequently become major intervention sites. However, empirical evidence supporting the effectiveness of school-based interventions against childhood obesity is of limited scope and unknown quality. The aim of this systematic review is to critically assess how researchers have characterized the school environment in determining its effect on childhood weight status in order to improve the quality and consistency of research in this area. We conducted a narrative review with a systematic search of the literature in line with PRISMA guidelines (2009). Original peer-reviewed research articles in English were searched from Medline, EMBASE, CENTRAL, CINAHL and PsycINFO databases from earliest record to January 2014. We included empirical research that reported at least one measure of the primary/elementary school environment and its relationship with at least one objective adiposity-related variable for students aged 4–12 years. Two authors independently extracted data on study design, school-level factors, student weight status, type of analysis and effect.

**Results:**

Five studies met the inclusion criteria. Each study targeted different parts of the school environment and findings across the studies were not comparable. The instruments used to collect school-level data report no validity or reliability testing.

**Conclusions:**

Our review shows that researchers have used instruments of unknown quality to test if the school environment is a determinant of childhood obesity, which raises broader questions about the impact that schools can play in obesity prevention.

## Background

Obesity has risen dramatically since the 1980s in most developed nations [1]. The disease is acquired from a sustained positive energy imbalance, with poor eating and activity behaviors, genetic, behavioral, environmental, and economic factors contributing to its development [2]. Obesity is associated with an increased risk of diabetes, cardiovascular disease and certain cancers, as well as other negative health and social outcomes [3,4].

Governments have focused prevention efforts on improved dietary and activity choices. These programmes particularly target children and adolescents, as obesity in youth is a robust predictor of adult obesity [5]. Schools provide unparalleled access to children and have therefore become the preferred setting for prevention strategies in the past few decades [3]. Empirical evidence, however, supporting the effectiveness of school-based interventions against childhood obesity is limited and of unknown quality.

Schools are well-defined environments that are hypothesized to influence student health outcomes by means of both compositional (which people are found in a place) and contextual factors (the characteristics of a place) [6]. In 2011, the Cochrane Collaboration reviewed school-based interventions that aimed to prevent childhood obesity [7]. The review was not restricted to schools, but the majority of included studies were school-based (78%) and targeted children aged 6–12 years, which is primarily why we restricted this review to primary/elementary schools. The authors concluded that obesity prevention programmes reduced childhood adiposity despite a high level of observed heterogeneity among study outcomes (I^2^ = 82%). In addition, for those studies that reported successful outcomes against childhood obesity, the authors reported a great deal of uncertainty about the levels (school- or individual-level) at which these interventions were effective.

It is important that researchers distinguish between institutional- and individual-level influences. A commonly used research framework to help categorize different environmental components is the ANGELO (Analysis Grid for Environments Linked to Obesity) framework. It is a conceptual model that aims to help researchers better understand the ‘obesogenicity’ (measure of obese-promotion) of different environments, with a school recognized as a micro-environment. We have applied this framework to help categorize the school environmental factors found in this review into one of four pillars: the economic (what are the costs), physical (what is available), political (what are the rules) and socio-cultural (what are the attitudes and beliefs) elements [8].

There is no agreed approach on how to capture the impact of a particular environment on a health outcome, which brings serious limitations to our understanding of the effectiveness of environmental interventions. Reviews regarding the ‘school effect’ on student weight status continue to call for more evidence in order to draw reliable conclusions [9,10]. Yet the evidence base cannot be improved until there is clarity on how researchers have characterized the school environment in determining its effect on obesity among students. Many research studies have included an environmental component as part of their intervention, but there continues to be a lack of consistency around how the environmental influences are measured. This is likely to continue until we have greater clarity on the ways in which researchers have characterized the environment, in this case schools, in determining its effect on weight status.

Therefore the aim of this systematic review is to critically assess how researchers have characterized the school environment in determining its effect on childhood weight status, with the hope to improve the quality and consistency of research in this area.

## Methods

We searched five electronic databases (Medline, EMBASE, CENTRAL, CINAHL, and PsycINFO) following the Preferred Reporting Items for Systematic Reviews and Meta-Analyses (PRISMA) review process [11]. The review protocol and questions were also registered prior to analysis on the International Prospective Register of Systematic Reviews (PROSPERO) (Registration No. CRD42014008829). No ethics approval was sought for this systematic review of the literature as no primary data collection took place. Inclusion criteria were English-language primary research articles that captured at least one primary/elementary school-level measure of the environment and at least one objective adiposity-related variable for students aged 4–12 years. The school environment is defined by the authors of the study as either a primary or elementary school, while a school-level measure refers to a school environmental factor (e.g. healthy eating policy, subsidized meals, vending machines, etc.) that has been researched in relation to student weight status.

Terms were developed using database keywords with variations of the following: school* OR ‘school environment’ OR ‘health? promoting schools’ OR ‘whole?school’ AND child* OR adolescen* OR student* OR pupil* OR ‘school? children’ OR youth OR teen* AND obes* OR overweight OR ‘body mass index’ OR BMI OR adiposity OR per?cent body fat OR skin? fold thickness OR ‘abdominal obesity’ OR ‘central adiposity’ OR ‘waist circumference’.

The exclusion criteria were developed and applied by two researchers, with 100% agreement achieved on final articles. A third reviewer helped to resolve any disagreements concerning the exclusion criteria. Articles were excluded if they were (i) not published in the peer-reviewed literature; (ii) focused on children with previous conditions or morbidities; (iii) not based within the primary/elementary school setting; (iv) did not collect school-level outcome or exposure data; (v) did not collect an objective measure of student weight status; and, (vi) did not report the school environment’s effect on student weight status independent of any intervention being trialed.

### Quality assessment

This review was not restricted to any particular study design and as such we considered the Newcastle-Ottawa Scale (NOS) most suitable for assessing the quality of each article included in our review, as NOS provides a quality assessment for multiple designs. NOS implements a ‘star system’ to judge the studies based on three broad perspectives: the selection of the study groups; the comparability of the groups; and, the ascertainment of either the exposure or outcome of interest [12]. We modified the NOS slightly to involve those quality assessment (QA) items relevant to the studies included in this review, which in the end were only observational and cross-sectional. The five specific quality items assessed were (i) the presence of a clear statement of the study aims; (ii) the representativeness of study participants; (iii) a clear description of the sample; (iv) the method of ascertainment for school-level exposures; and, (v) the type of obesity measurement guidelines followed. The NOS ‘star system’ provides a score on a 9-point scale ranging from a highest-quality paper (score = 9) to lowest quality (score = 1).

### Synthesis of literature

The initial review of the studies identified that a meta-analysis was not possible and so a narrative synthesis of the literature was conducted to collate and summarize the results of the studies. To assist our analysis and abridge transparency, we used the ANGELO framework to categorize the school-level evidence. In the end, a total of five articles were included in our narrative synthesis [13-17]. These studies were included because the authors investigated the impact of school-level factors on objectively-measured student weight status. This is an important distinction as this review is not interested in the ‘school effect’ once it has been manipulated or changed, instead our review looks strictly at whether or not there is a ‘school effect’ independent of any intervention being trialed.

## Results

**Figure 1:**
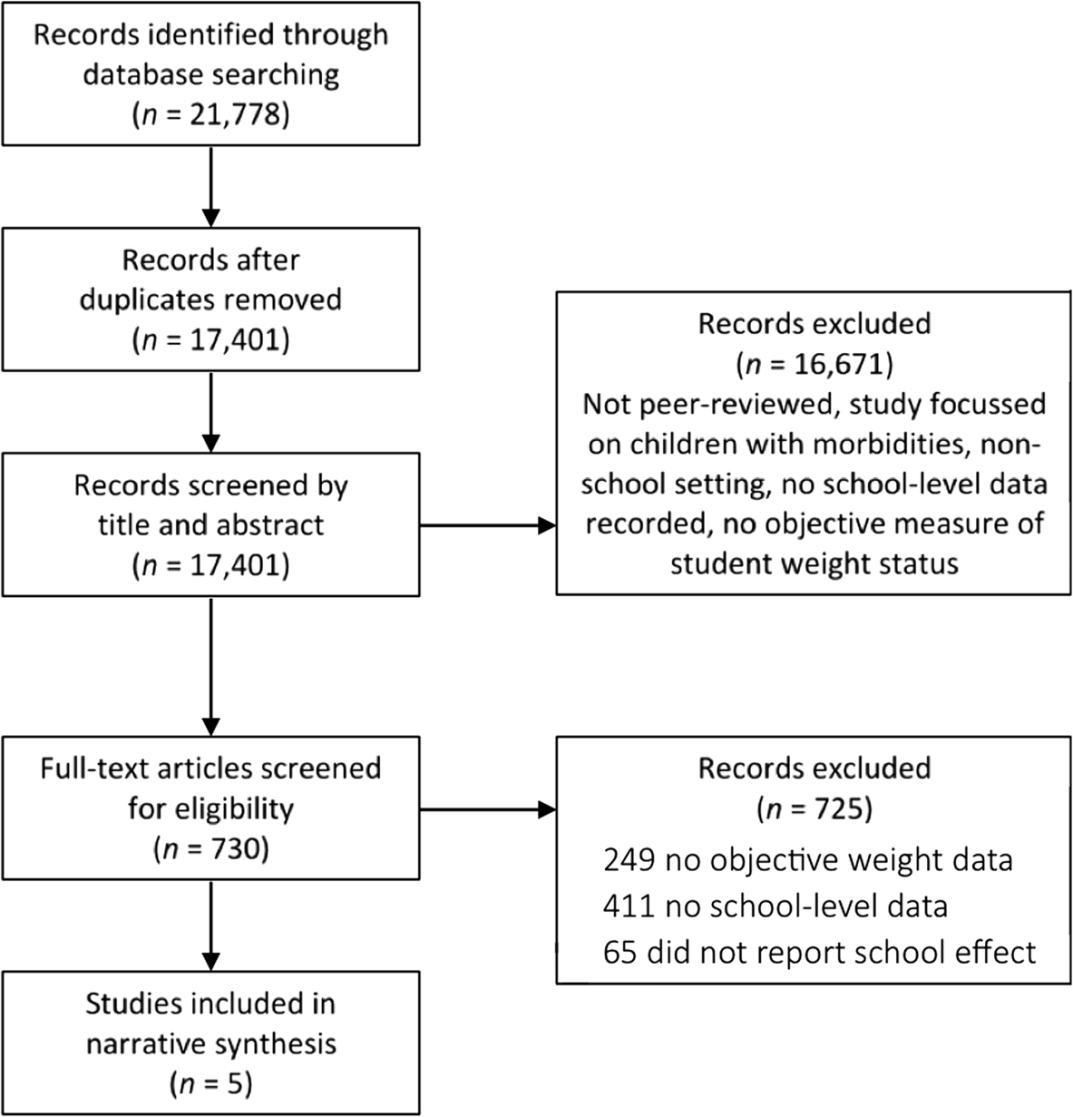
**Search strategy based on PRISMA guidelines** **[** 11 **]** **.**

Our systematic search of the literature identified 21,778 potential articles, including 4,377 duplicates (Figure 1). We found 730 articles that potentially met all criteria as a result of title and abstract screening. Of these, 725 did not meet the inclusion criteria, with the vast majority of articles focused only on certain intermediary factors (such as physical activity levels and dietary intake) without any objective measure of student adiposity, or they did not report any school-level data.

Of note, sixty-five articles were excluded in our narrative synthesis despite having the data required to analyze the effect of school-level factors on student weight status. These articles were focused on either the differences between cases and controls and/or the effect of a specific intervention being trialed; authors have thus not reported on the relationship between environmental factors and obesity status among students.

### Main study characteristics

All five studies included were cross-sectional (Table 1). Four out the five studies were conducted in North America and the other in the United Kingdom. We found variation in the quality of each study, with only one considered strong (QA: 7–9). All were based within the primary/elementary school setting as defined by the authors. Two studies were focused on Year 5 students and the other three presented results for combined year levels. We found studies had investigated a wide range of school- level factors in relation to its effect on student weight status. Studies were focused on either the food or activity environment, with one investigating both intermediaries.

**Table 1:** **Summary of study characteristics**

**Study/location**	**Study design/sample**	**Outcome of interest**	**Exposure measure used**			**School exposure measure and reported association(s) with student weight outcome (95% CI) (^ = p-value less than 0.05)**	**QA**
			**Method of data collection**	**Validity/reliability**	**ANGELO framework**		
Veugelers & Fitzgerald, 2005 [13]/Canada	Cross-sectional/5,200 Yr 5 students from 228 schools	Body Mass Index (BMI)	A written survey was completed by the school principal on the presence of healthy menu alternatives.	Not reported	A *Policy*-related factor was researched (1)	1. *School provided healthy menu alternatives:* Overweight = OR 0.91 (0.77, 1.09) Obesity = OR 0.85 (0.63, 1.15)	5
Fox *et al.* 2009 [14]/US	Cross-sectional/2,228 Yr 1–12 students from 287 schools	BMI (*obesity* only)	A written survey was completed by a foodservice manager about the frequency and type of foods made available in the cafeteria.	Not reported	Policy (2–6)	2. *Low-nutrient, energy-dense foods available* = OR 1.09 (0.57 – 2.08)^	8
						3. *Whole or 2% milk offered* = OR 1.17 (0.75 – 1.82)^	
						4. *Fresh fruit/raw vegetables not offered daily* = OR 1.13 (0.73 – 1.75)^	
						5. *French fries/ similar products offered regularly* = OR 2.70 (1.58 – 4.62)^	
						6. *Dessert offered more than once per week* = OR 1.78 (1.13 – 2.80)^	
Harrison *et al.* 2011 [15]/UK	Cross-sectional/1,725 Yr 5 students from 92 schools	Fat Mass Index (FMI)	A written survey was completed by a ‘head teacher’ about school policies.	Not reported	Physical (7)	7. *Lower FMI was found in girls attending schools with more pupils in their age group*^ (interquartile analysis)	4
					Policy(8–11)		
					Economic (12)		
			A ‘trained assessor’ completed an audit of school grounds.			8. *Better cycle support was associated with higher FMI in girls*^ (interquartile analysis)	
			Local council provided general information.			9. *Higher FMI was associated with boys who were allowed to eat any foods at break-time*^ (interquartile analysis)	
						**Insignificant findings not reported for:**	
						10. *Food-related learning*	
						11. *UK Govt ‘healthy school programme’*	
						12. *Free school meals*.	
Rundle *et al.* 2012 [16]/US	Cross-sectional/624,204 Yr K-12 students from 1,276 schools	BMI	Data were extracted from the New York City Department of Education enrolment database.	Not reported	Economic (13) & Socio-cultural (14)	13. *Students received free or reduced-price lunches:* Overweight = OR 1.05 (1.00, 1.08)^ and Obesity = OR 1.13 (1.10, 1.18)^	5
						**Insignificant findings not reported for:**	
						14. *Ethnicity of students in school*.	
Leatherdale, 2013 [17]/Canada	Cross-sectional/2,331 Yr 1–4 students from 30 schools	BMI (*over-weight* only)	A written survey was completed by the ‘senior administrator most knowledgeable about school policies and practices’.	Not reported	Physical (15–16)	15. *Moderate level of student access to a variety of facilities on and off school grounds during school hours* = OR 0.39 (0.16, 0.92)^	1
					Policy (17–21)		
					Socio-cultural (22–25)		
			A school built environment survey was completed by a ‘trained assessor’ using the ‘Environmental Points of Interest’ tool.			16. *Good level of student access to a variety of facilities on and off school grounds during school hours* = OR 0.32 (0.12, 0.86)^	
						**Insignificant findings not reported for:**	
						17. *PA used as reward*	
						18. *Good PA transport to and from school*	
						19. *Good implementation of daily PA*	
						20. *Good amount of daily PA*	
						21. *Good training of PA teachers*	
						22. *Good consistency of intramural PA*	
						23. *Good incorporation of PA into other subjects*	
						24. *Good community feedback on school PA*	
						25. *Good PA promotion by teachers*	

^Results were reported as statistically significant (p-value less than 0.05).

The application of the ANGELO framework greatly assisted in our analysis of school-level factors, which were grouped into one of four environmental elements: physical, policy, economic, and socio-cultural. The presence of policy-related elements were most common among studies, with one school-level factor related to policy implementation. Socio-cultural elements of the school environment were the second most common school-level factor, while a small amount investigated the physical and economic elements within the school setting.

### Quantitative findings

Studies reported a variety of food- and activity-related factors that were associated with student weight status and statistically significant. These findings, however, represent only a small proportion of the number and variety of school-level factors investigated. Fox *et al.* reported the availability of french fries and desserts in the school cafeteria as associated with obesity among primary school students [14]. Harrison *et al.* reported lower FMI rates in girls attending schools with more pupils in their age group, whereas higher FMI was associated with higher cycle provision among girls. Boys were reported to have a higher FMI if allowed to eat any foods at break-time [15]. Rundle *et al.* reported obesity to be associated with students who received free or reduced-price lunches [16].

Four studies reported school-to-student associations that were statistically significant (*p* <0.05) [14-17]. All studies used a regression type of analysis, with two applying a multi-level model to separate compositional and contextual influences. Three studies used Body Mass Index (BMI) as their primary outcome measure in line with International Obesity Task Force (IOTF) guidelines [13,16-18], while Fox *et al.* applied guidelines recommended by the US Centers for Disease Control and Prevention (CDC) [14,19]. Harrison *et al*. used Fat Mass Index (FMI) in their model, which was derived from foot-to-foot bioelectrical impedance values [15].

All authors acknowledged the limitations of a cross-sectional study design and that it is not possible to conclusively attribute associations between characteristics of the school environment and student BMI. Harrison *et al.* stated that foot-to-foot bioelectrical impedance measures of body fat do not measure the composition of the upper body. Rundle *et al.* reported that the exclusion of private schools was a limitation. Four studies were limited by focusing on school-level factors that concerned just one side of the energy intake/expenditure pathway. We found much inconsistency in the number and type of variables adjusted for in final models. No studies reported adjustments for existing school-based programmes present at the time of data collection.

Despite authors reporting very few, if any, limitations to the instrument(s) they used to collect school-level data, no study reported the validity or reliability of these instruments. Each study collected their school-level data from a different source, either from school staff or their own data collectors. We found that the authors offered little information about the theories behind their approach to school-level data collection.

## Discussion

The five articles included in this review found poor-quality evidence supportive of a relationship between schools and student weight status. Evidence was collected using a variety of instruments that had no validity or reliability testing, nor with any references to their development or theoretical framework.

A total of 65 peer-reviewed publications were identified that had the data needed to investigate the relationship between school- and student-level factors. However, these authors elected to either not report the school-to-student association at baseline, or they simply were not concerned with the ‘school effect’ beyond the effect of their intervention.

There are important challenges to consider in measuring the ‘school effect’ , if present; multi-level modeling offers one such solution in helping to unpick specific factors associated with student weight status at the school-level. Our review was not restricted by type of study design, yet all five studies included were limited by a cross-sectional approach to the data set. This would appear at first to make sense considering our exclusion criteria, but intervention studies could have reported the ‘school effect’ at baseline and therefore they would have been included. There was little consistency found in the sampling of primary school students and the types of outcome data used to measure student weight. Only one study was considered to be of strong research quality. Authors were either focused on the school food environment or the activity environment when investigating the school’s effect on student weight status, while one study looked at both determinants.

We grouped school-level measures in accordance with the ANGELO framework to improve transparency of the environmental findings. We found studies had investigated a wide range of school-level factors, but there was a lack of consistency between studies. In their analysis, authors tended to either isolate certain parts of the school environment or capture multiple components simultaneously. Different environmental elements may have greater influence on student weight status than others, while certain combinations may also be consequential. These methodological issues should be considered in the study design phase of future research.

School-level variables that related to school policies (what are the rules) were the most common element investigated by researchers, but there was only one case where the study looked at policy implementation. It is potentially more useful to know if a policy is acted upon or neglected. The most surprising result, however, was the lack of exploration by researchers into the economic environment (what are the costs) within schools. It is well established that pricing can influence dietary choices and this is an area of the school environment that should be further investigated in relation to student weight status.

Considering the objective nature of school-level factors related to the physical environment (what is available), it was surprising that more research had not been done here. An objective measure within this context is an estimate of a school-level factor that another researcher could also attain, such as: oval size, food pricing, and the availability of equipment. These data types are important for comparative research and statistical modeling moving forward – particularly with regard to improving the reliability of instruments – but this should not deter efforts to also capture various socio-cultural factors (what are the attitudes and beliefs) within a school setting.

## Conclusions

This review has highlighted a lack of evidence showing that the school environment is a determinant of childhood obesity and that researchers have used research instruments of unknown quality to test for this relationship. Studies also face the challenge of measuring the school effect, if present, on student weight status. In future, we recommend that researchers aim to include objective measurements, wherever feasible, at both the school- and student-level, and this research should also include school- and student-level factors relevant to both the food and activity environment. These types of data will support statistical techniques that can separate compositional and contextual factors. These studies would also be enhanced with follow-up data. Research of this ilk should aim to identify modifiable risk factors and specific school-level features that are amenable to programmatic and policy intervention.

## Abbreviations

ANGELO: Analysis Grid for Environments Linked to Obesity
PRISMA: Preferred Reporting Items for Systematic Reviews and Meta-Analyses
PROSPERO: International Prospective Register of Systematic Reviews
NOS: Newcastle-Ottawa Scale
QA: Quality Assessment
BMI: Body mass index
FMI: Fat mass index
OR: Odds ratio
CI: Confidence intervals
IOTF: International Obesity Task Force
US: United States
CDC: Centers for Disease Control and Prevention
